# Editorial: Spatiotemporal & AI trends in neuroscience, neuroimaging, and neurooncology

**DOI:** 10.3389/fnimg.2025.1716335

**Published:** 2025-10-29

**Authors:** Alessandro Crimi, Spyridon Bakas

**Affiliations:** ^1^Department of Computer Science, AGH University of Kraków, Krakow, Poland; ^2^Department of Pathology and Laboratory Medicine, Indiana University School of Medicine, Indianapolis, IN, United States; ^3^Indiana University Melvin and Bren Simon Comprehensive Cancer Center, Indianapolis, IN, United States; ^4^Department of Neurological Surgery, Indiana University School of Medicine, Indianapolis, IN, United States; ^5^Department of Radiology and Imaging Sciences, Indiana University School of Medicine, Indianapolis, IN, United States; ^6^Department of Biostatistics and Health Data Science, Indiana University School of Medicine, Indianapolis, IN, United States; ^7^Department of Computer Science, Luddy School of Informatics, Computing, and Engineering, Indiana University, Indianapolis, IN, United States

**Keywords:** spatiotemporal modeling, artificial intelligence, deep learning, neuroimaging, neurooncology, precision medicine, brain networks, biomarkers

Longitudinal brain changes over years, or even days, can reflect changes and insights that can be captured and studied with data science. In this context, this special issue focuses on neuroscience, neurooncology, and neuroimaging. Powerful paradigms, such as advanced artificial intelligence (AI) (Zhang et al., 2019) and spatio-temporal modeling, either biophysical or biomechanical (Mang et al., 2020), have been contributing in transformational ways. This is changing our understanding of brain activity, disease diagnosis, and response to treatment.

It is indisputable that brain function is spatio-temporal. Indeed, we use different methods to quantify different structural and functional aspects of the brain: Diffusion tensor imaging (DTI) maps the structural pathways that support communication, electroencephalography (EEG) magnetoencephalography (MEG) record rapid electrical and magnetic fluctuations, and functional magnetic resonance imaging (fMRI) records slow hemodynamic correlating to neural activity throughout the brain. We need all those modalities to grasp a minimum of understanding of the complex responses of the neurological system. As evidenced by the work in this issue, novel techniques are emerging to model brain activity as a continuous, high-dimensional spatio-temporal process. Dynamic Functional Connectivity (dFC) analyses reveal how networks reconfigure according to timescales (Preti et al., 2017), while graph neural networks (GNNs) are being used to model the propagation of pathological activity in epilepsy or the spread of neurodegeneration (Li et al., 2022).

The rise of AI, especially deep learning, is the driving force behind this revolution. Without the need for strong *a priori* hypotheses, these models are uniquely suited to reveal complex, non-linear patterns hidden within massive neuroimaging datasets. This enables sophisticated AI architectures to handle multi-modal data integration/fusion, combining MRI, metabolic, histology, genomics, and clinical records, to create a more comprehensive view of the patient.

Nowhere is the impact of this spatiotemporal-AI synergy more impressive than in neurooncology. Management of brain tumors requires precise characterization (e.g., imaging genotyping), delineation of invasive margins, differentiation of treatment effects from true progressive disease (i.e., tumor recurrence), and prediction of patient overall survival and treatment response. All of these are complex spatio-temporal problems. For example, to predict the grade of glioma and molecular markers, such as the status of IDH mutation (Pytlarz et al., 2024), advanced AI models have been applied to multiparametric MRI, allowing non-invasive characterization and personalized treatment planning (Akbari et al., 2020). Spatiotemporal models can track tumor growth trajectories (Mang et al., 2020) and simulate the effects of radiotherapy or resection recovery (Falcó-Roget et al., 2024), moving toward truly personalized treatment planning.

To comprehend trajectories of degeneration or recovery, longitudinal studies with many time-points spread over years have been increasingly useful. Comprehensive multiyear studies such as the Alzheimer's Disease Neuroimaging Initiative (ADNI) (Mueller et al., 2005), which provide multimodal longitudinal data sets that track individuals from healthy aging through mild cognitive impairment to Alzheimer's dementia, have been extremely beneficial to this effort. Instead of relying on only single-time point cross-sectional images, AI models can now use these longitudinal data to assess the temporal progression of the disease. Recurrent neural networks (RNNs) and latent variable models are being trained to predict future rates of cortical thinning, hippocampal atrophy, and cognitive decline based on an individual's initial scan and clinical profile (Li and Fan, 2019). These techniques bring us closer to developing personalized disease development timelines, identifying pivotal moments in pathology, and evaluating the efficacy of treatments by detecting subtle year-over-year changes that are imperceptible to the naked eye.

The research presented in this Research Topic offers an engaging look at a rapidly evolving topic. We are transitioning from static snapshots to dynamic, predictive models of brain health and disease through the combination of spatio-temporal analytics and AI.

In order to speed up the process from the bench to the bedside, we anticipate that this will encourage further innovation and cooperation between the fields of neuroscientific, clinical, and computational science in the future.
